# Bta-miR-146b promotes infectious bovine rhinotracheitis virus replication and inhibits type I interferon expression via targeting IRAK1

**DOI:** 10.3389/fcimb.2026.1718649

**Published:** 2026-01-23

**Authors:** Siping Zhu, Hong Li, Chihuan Li, Xintong Zhu, Chao Ren, Qiumei Shi, Tonglei Wu, Guangping Gao, Yonghui Li, Li Chen, Zhiqiang Zhang

**Affiliations:** 1 Hebei Key Laboratory of Preventive Veterinary Medicine, Hebei Normal University of Science & Technology, Qinhuangdao, China; 2 Department of Dermatology, The Second Hospital of Qinhuangdao, Qinhuangdao, China

**Keywords:** bta-miR-146b, IBRV, IRAK1, miRNA, type I IFN

## Abstract

**Background:**

Infectious bovine rhinotracheitis virus (IBRV) is a primary pathogen causing bovine respiratory disease syndrome. This virus can cause rhinotracheitis and vaginitis in cattle, resulting in high mortality and posing a serious threat to bovine production. MicroRNAs (miRNAs), a class of regulatory non-coding small RNAs, can modulate viral replication by influencing host immune responses. However, reports on the association between host miRNAs and IBRV infection are limited.

**Methods:**

In this study, we screened differentially expressed miRNAs in MDBK cells after IBRV infection and determined that the expression of bta-miR-146b was significantly increased. We investigated the effects of bta-miR-146b on IBRV replication and its underlying molecular mechanisms using molecular biological techniques such as luciferase activity assays, Western Blot, and qRT-PCR, together with bioinformatics approaches.

**Results:**

We found that bta-miR-146b expression was up-regulated in IBRV-infected MDBK cells. Furthermore, transfection with bta-miR-146b mimics promoted IBRV replication in MDBK cells, whereas transfection with bta-miR-146b inhibitors inhibited IBRV replication, indicating that bta-miR-146b is a pro-infection factor. Additional studies showed that bta-miR-146b mimics inhibited type I interferon expression in MDBK cells, whereas its inhibitors enhanced it. Moreover, we identified IRAK1 as a direct target of bta-miR-146b and found that silencing IRAK1 expression rescued the effects of bta-miR-146b on viral replication and type I interferon expression.

**Conclusion:**

These results suggest that bta-miR-146b regulates type I interferon expression and IBRV replication in MDBK cells by targeting IRAK1, and plays a key role in IBRV infection.

## 1 Introduction

Infectious Bovine Rhinotracheitis Virus (IBRV), also known as bovine herpesvirus type 1 (BoHV-1), is a major etiologic agent of bovine respiratory disease syndrome. This virus belongs to the Herpesviridae family and has the ability to establish latent infections in the host (Ostler and Jones, 2023). IBRV can be transmitted through multiple routes, causing diverse clinical signs in cattle, such as acute rhinitis, conjunctivitis, pneumonia, abortion, and reproductive disorders. Besides the direct damage it caused, IBRV can trigger host immunosuppression, leading to subsequent infections or co-infections by other pathogens. These complications cause significant economic losses and pose a continuous threat to the sustainability of the global bovine industry (Muylkens et al., 2007).

IFN-I play critical roles in the host’s antiviral immune defense. When a viral infection occurs, the host cell detects viral pathogen-associated molecular patterns through pattern recognition receptors. This recognization initiates the IFN signaling pathway and activates the expression of type I interferons, which exert antiviral effects by inhibiting viral replication (McNab et al., 2015). However, viruses can evade this defense mechanism through various strategies. Many viral proteins can directly interact with host proteins to interfere with type I interferon expression or recruit the ubiquitin proteasome system to destabilize proteins that are important for IFN responses (McInerney and Karlsson, 2009; Men et al., 2023; Zhang et al., 2023). In addition, some viruses regulate type I interferon expression by modulating host miRNAs (Li et al., 2020; Takahashi and Ui-Tei, 2020; Qian et al., 2022). IBRV was reported to be able to interfere with type I interferon expression through multiple pathways. Its gE protein can suppress type I interferon expression by promoting MAVS ubiquitination and interfering with the interaction between IRF3 and CBP/p300 (Liu et al., 2023). The tegument protein UL3 of IBRV has the ability suppresses antiviral IFN-I signaling by targeting STING (Sun et al., 2024). Besides, IBRV has ability to encodes several proteins, bICP0, bICP27, gG, UL49.5, and VP8, which interfere with key antiviral innate immune responses in the absence of other viral genes (Jones, 2019).

MicroRNAs (miRNAs) are a class of small non-coding RNA molecules of approximately 22 nucleotides in length, capable of post-transcriptional regulation of target gene expression, and widely involved in biological processes such as cell differentiation, immune response and pathogen-host interactions (Saliminejad et al., 2019). Although it is well established that viruses can modulate host immune response using miRNAs (Nejad et al., 2018), the reports of its relevance in IBRV infection are limited. It has been reported that IBRV can encode at least 10 miRNAs involved in the regulation of viral replication, while host encoded bta-miR-2890 has been reported to up-regulate JAK-STAT pathway to inhibit BoHV-1 replication by targeting viral gene UL41 (Ma et al., 2020).

In this study, we screened differentially expressed miRNAs in MDBK cells after IBRV infection and determined that miR-146b can promote viral replication by targeting IRAK1, demonstrating that miR-146b plays a key role in IBRV infection.

## 2 Materials and methods

### 2.1 Cells and virus

MDBK cells and 293T cells were purchased from BeNa Culture Collection (Shanghai, China) and cultured in Dulbecco’s modified Eagle medium (DMEM, Gibco, USA) supplemented with 10% fetal bovine serum (Gibco, USA) and 1% penicillin-streptomycin solution (Solarbio, China). The cultures were incubated in an incubator at 37°C under an atmosphere of 5% CO_2_. The IBRV strain HBCL-1 was isolated from calves with rhinotracheitis and stored in the Laboratory of Animal Infectious Diseases, Hebei Normal University of Science & Technology.

### 2.2 Sequences of miRNA mimics or inhibitors

Mimics and inhibitors for miRNA were synthesized by GenePharma (Shanghai, China). The gene sequences are listed as below (**Table 1**).

**Table 1:** miRNA primer sequences.

Primers	Sequences (5′-3′)	Antisequences (5′-3′)
miR-146b mimics	UGAGAACUGAAUUCCAUAGGCUGU	AGCCUAUGGAAUUCAGUUCUCAUU
mir-146b negative control	UUCUCCGAACGUGUCACGUTT	ACGUGACACGUUCGGAGAATT
miR-146b inhibitor	ACAGCCUAUGGAAUUCAGUUCUCA	
mir-146b inhibitor negative control	CAGUACUUUUGUGUAGUACAA	

### 2.3 Deep sequencing and miRNA target prediction

Deep sequencing was performed by Shanghai Meiji Biotechnology Co., Ltd. (Shanghai, China) on MDBK cells infected with mock or IBRV at an MOI of 1 for 24 h. miRNA targets in host cells were predicted by TargetScan (version 3.1). We preferentially selected 8mer/7mer-m8/7mer-A1 loci, combined with CWCS ≤ -0.2 and PCT > 0 screens.

### 2.4 RNA isolation and quantitative real-time PCR analysis

Total RNA was extracted from MDBK cells using a Trizol kit according to the manufacturer’s instructions (Invitrogen, USA). RNA was reverse transcribed into cDNA using the ReverTra Ace^®^ qPCR RT Master Mix with gDNA Remover kit (Toyobo, Japan), and qRT-PCR was performed using the Ultra SYBR Mixture (Cwbio, China). miRNAs were extracted from MDBK cells using an E.Z.N.A.PF Micro RNA Kit (Omega Bio-Tek, USA). miRNA expression was analyzed using the Hairpin-it miRNAs RT-PCR Quantitation Kit (GenePharma, China). The specific primers used for qPCR in this study are listed in **Table 2**.

**Table 2:** miRNA primer sequences.

Primers	Nucleotide sequences (5′-3′)
gD-F	CGCCACGGTCATATGGTACA
gD-R	TAGCGGCAGTACCCAAAGTG
IRAK1-F	ATGGCCGGGGGGCCGGGC
IRAK1-R	TCAGCTCCGAAACTCGTCTCTCTCTT
IFN-α-F	GTGAGGAAATACTTCCACAGACTCACT
IFN-α-R	TGAGGAAGAGAAGGCTCTCATGA
IFNβ-F	CCTGTGCCTGATTTCATCATGA
IFNβ-R	GCAAGCTGTAGCTCCTGGAAAG
p65-F	CCACAACACAATGCGCTCTG
p65-R	AACTCAGCGGCGTCGATG
IRF3-F	CCGCCGATCCCTGAGAGTG
IRF3-R	AAGCCTAGGCCTTCTGGGC
bta-miR-146b-F	TTAGCCCGTGAGAACTGAATTCCA
bta-miR-146b-R	TATCCTTCTTCACGACTCCTTCAC
GAPDH -F	GATTGTCAGCAATGCCTCCT
GAPDH-R	GGTCATAAGTCCCTCCACGA
U6-F	CAGCACATATACTAAAATTGGAACG
U6-R	ACGAATTTGCGTGTCATCC

### 2.5 Western blot analysis

For detection of gD or IRAK1 expression in MDBK cells, cell lysates were prepared using a non-denaturing lysis buffer (50 nM Tris-HCl, pH 8.0, 150 nM NaCl, 1% TritonX-100, 5 nM EDTA, 10% glycerol, 10 nM dithiothreitol, 1×complete cocktail protease inhibitor). The cell lysates were mixed with 4×SDS loading buffer and boiled for 10 min. The protein samples were fractionated by electrophoresis on 12% SDS-polyacrylamide gels, and resolved proteins were transferred onto polyvinylidene difluoride (PVDF) membranes. After blocking with 5% skimmed milk, the membranes were incubated with anti-gD, anti-IRAK1 or anti-GAPDH antibody, followed by HRP-conjugated anti-Mouse or anti-Rabbit secondary antibody. Blots were developed using an enhanced chemiluminesence (ECL) kit per the manufacturer’s instruction.

The protein marker used in this experiment was purchased from Shanghai Epizyme Biomedical Technology Co., Ltd (Shanghai, China), catalog number WJ102. The anti-gD monoclonal antibody was kindly provided by Prof. Wenxiao Liu, Beijing Academy of Agriculture and Forestry. Rabbit-derived anti-bovine IRAK1 polyantibody was purchased from Pro-Tech Biological Research Center Limited (Jiangsu, China).

### 2.6 Transfection of miRNA mimics or inhibitors for the measurement of IBRV growth

MDBK cells were seeded in 12-well plates for 12 h and then transfected with miRNA mimics or inhibitors using INTERFERin transfection reagent (PolyPlus, France). After 24 h of transfection, cells were infected with IBRV. Then cells were collected for virus titration and gD expression by western blotting or qPCR analysis at 24 h after IBRV infection.

### 2.7 Determination of IBRV growth in MDBK cells

Normal MDBK cells or MDBK cells transfected with bta-miR-146b mimics, bta-miR-146b inhibitor or controls were infected with IBRV at an MOI of 1. The cell cultures were collected at different time points of infection (12, 24, 48 h) and centrifuged at 8000 rpm/min for 10 min after three repeated freeze-thawing. The viral content in the supernatants was determined using a 50% tissue culture infectious dose (TCID_50_). Briefly, MDBK cells were seeded in 96-well plates with approximately 5 × 10^5^ cells/mL. The virus solution collected was serially diluted 10-fold with DMEM medium and subsequently added to 96-well plates at a dose of 100 μL per well. Cells were cultured for 7 days at 37°C under 5% CO_2_, and tissue culture wells with a cytopathic effect (CPE) were determined as positive. Viral titers were calculated according to methods described in the literature (Reed and Muench, 1938).

### 2.8 Luciferase activity assays

A 500 bp IRAK1 gene fragment around the predicted bta-miR-146b target site was amplified and inserted into the GP-mirGLO dual-luciferase reporter vector (GenePharma, China) through the *Sac*I and *Xho*I cleavage sites to construct the recombinant luciferase plasmid GP-mirGLO-target-IRAK1-WT. The mutant plasmid GP-mirGLO-target-IRAK1-MUT was also constructed with binding site mutations by substituting the target sequence GUUCUCA for GUAGAGA. The 293T cells were seeded in 12-well plates and cultured overnight, and then co-transfected with GP-mirGLO-target-IRAK1-WT or GP-mirGLO-target-IRAK1-MUT and bta-miR-146b mimics or controls using INTERFERin transfection reagent. After 48 h post-transfection, luciferase activity assays were performed using a Luc-Pair™ Duo-Luciferase Assay Kit 2.0 (TransGen, China) according to the instructions. Firefly luciferase activity was normalized by sea kidney luciferase activity.

### 2.9 Knockdown of IRAK1 by RNA interference

The siRNAs for IRAK1 knockdown were designed and synthesized by GenePharma (Shanghai, China), a total of four siRNA sequences were designed and the sequences were illustrated in **Table 3**. MDBK cells were seeded in 12-well plates and cultured for 12 h, and then transfected with siRNAs or controls using INTERFERin transfection reagent. The cells were harvested after 24 h for further analysis.

**Table 3:** siRNA primer sequences.

Primers	Sequences (5′-3′)	Antisequences (5′-3′)
IRAK1-Bos-89	GUCAUGUGCCGCUUCUACATT	UGUAGAAGCGGCACAUGACTT
IRAK1-Bos-1049	GUCCUUCUGGAUGAGAGACTT	GUCUCUCAUCCAGAAGGACTT
IRAK1-Bos-1303	GCCCAAGUAUCUGAAAGACTT	GUCUUUCAGAUACUUGGGCTT
IRAK1-Bos-1783	GAGCGUGUCUGACCUCUCUTT	AGAGAGGUCAGACACGCUCTT
IRAK1-negative control	UUCUCCGAACGUGUCACGUTT	ACGUGACACGUUCGGAGAATT

### 2.10 Statistical analysis

Statistical analysis was performed using GraphPad Prism version 8.0, and t-tests were performed using one-way analysis of variance (ANOVA). Significant differences are marked with an asterisk (*), where *p < 0.05, **p < 0.01, and ***p < 0.001 indicate statistically significant differences in means.

## 3 Results

### 3.1 bta-miR-146b expression is upregulated during IBRV infection

**Figure 1:**
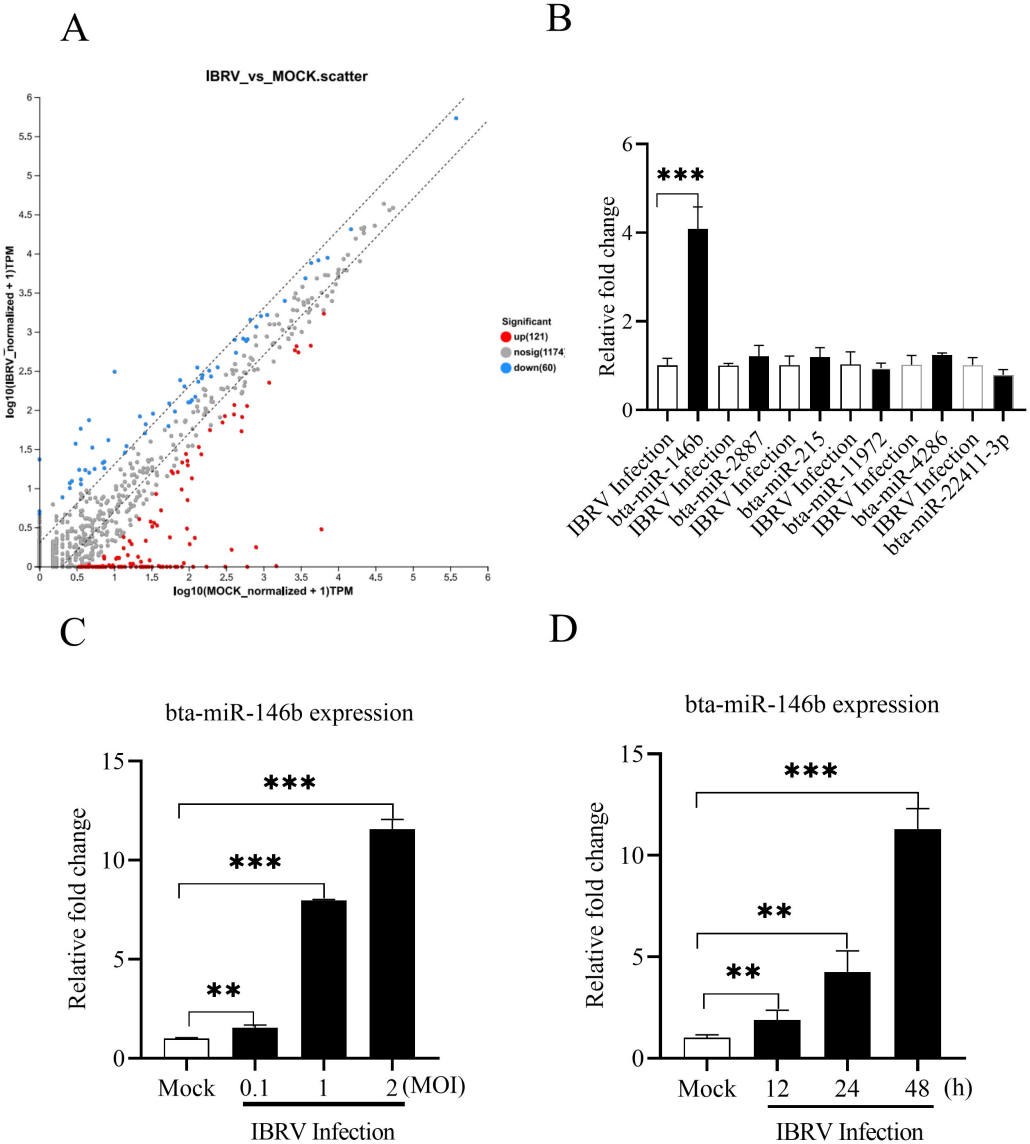
Assay of miRNAs in MDBK cells during IBRV infection. **(A)** Heatmap showing the expression profiles of miRNAs in MDBK cells with or without IBRV infection in deep sequencing. Red color represents increased miRNA expression and blue color represents decreased miRNA expression. **(B)** MDBK cells were infected with IBRV at an MOI of 1. Intracellular miRNAs were extracted at 24 h post-infection and expression of 6 miRNAs was determined by qPCR, and U6 expression was used as an internal control. **(C)** The expression of bta-miR-146b was examined in MDBK cells after infection with different doses of IBRV. MDBK cells were infected with IBRV at MOIs of 0.1, 1 and 2, respectively. Intracellular miRNAs were extracted 24 h after infection, and bta-miR-146b expression was assayed by qRT-PCR with U6 as an internal control. **(D)** Bta-miR-146b expression was assayed at different time points after IBRV infection. MDBK cells were infected with IBRV with an MOI of 1. Intracellular miRNAs were extracted at different time points (12, 24, 48 h) after IBRV infection, and bta-miR-146b expression was assayed by qRT-PCR with U6 as an internal control. Data represent the mean of three replicate experiments. (**p < 0.01, ***p < 0.001).

To investigate miRNA expression during IBRV infection, we performed deep sequencing to determine the expression changes of miRNAs in MDBK cells at 24 h post infection with IBRV. The results showed (**Figure 1A**) that a total of 181 miRNAs displayed altered expression after IBRV infection. Six miRNAs were picked for expression confirmation using the qPCR method, and the results showed that of these miRNAs, bta-miR-146b expression was significantly upregulated after IBRV infection (**Figure 1B**).

To further determine the effect of IBRV infection on the bta-miR-146b expression, we infected MDBK cells with different doses of IBRV and detected the expression of bta-miR-146b in the cells at 24 h post-infection. The results showed that the expression of bta-miR-146b in cells was significantly increased after IBRV infection in a dose-dependent manner (**Figure 1C**). We then infected MDBK cells with IBRV at a dose of MOI of 1 and detected the expression of bta-miR-146b at different time points after infection. The results showed that the expression of bta-miR-146b was also upregulated in the cells with a time-dependent manner (**Figure 1D**). These data suggest that IBRV infection can induce bta-miR-146b expression in host cells.

### 3.2 bta-miR-146b promotes IBRV replication in MDBK cells

**Figure 2:**
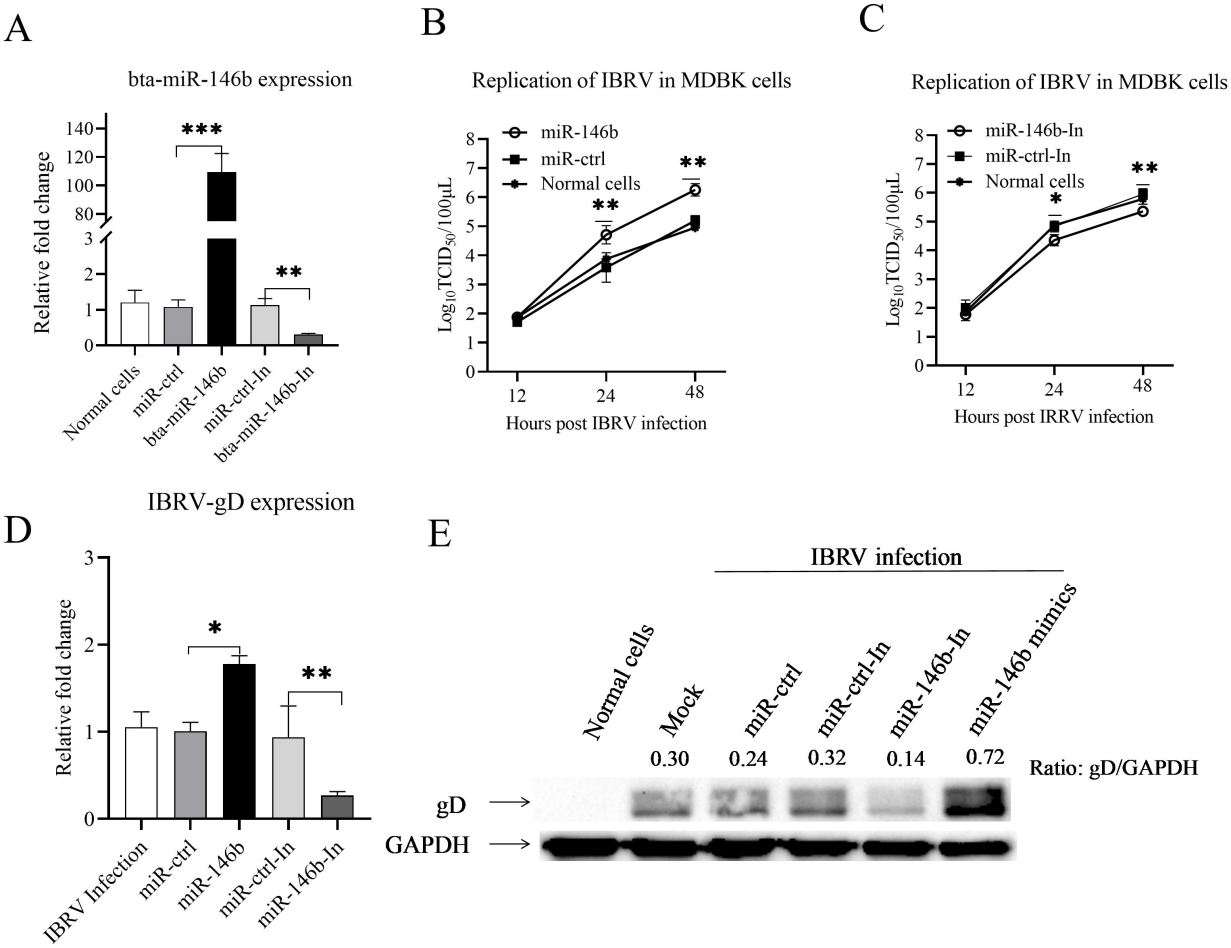
bta-miR-146b promotes IBRV replication in MDBK cells. **(A)** Measurement of bta-miR-146b expressions in cells transfected with bta-miR-146b mimics or bta-miR-146b inhibitor. MDBK cells were transfected with bta-miR-146b, miRNA control, bta-miR-146b inhibitor, or miRNA inhibitor control. Twenty-four hours post-transfection, cells were harvested for quantifying the expression of bta-miR-146b by qRT-PCR. **(B, C)** The effect of bta-miR-146b expression on IBRV replication was analyzed by the TCID_50_ assay. MDBK cells were transfected with miRNA control, miR-146b mimics, or miR-146b inhibitor, respectively. Twenty-four hours after transfection, cells were infected with IBRV at an MOI of 1. Viral load in cell cultures was determined at different time points after IBRV infection (12, 24, 48 h) using TCID_50_ assay. **(D)** Transfection of MDBK cells with bta-miR-146b affects gD gene expression. MDBK cells were transfected with 50 nM of bta-miR-146b mimics, inhibitor or controls for 24 h, and then MDBK cells were infected with IBRV with an MOI of 1. Total cellular RNA was extracted and the gD gene expression was detected by qPCR. GAPDH was used as an internal control. **(E)** Transfection of MDBK cells with bta-miR-146b affects gD protein expression. MDBK cells were transfected with 50 nM of bta-miR-146b mimics, inhibitor or controls for 24 h, and then MDBK cells were infected with IBRV with an MOI of 1. After 24 h of infection, cell lysates were prepared for western blot to detect gD protein expression, and endogenous GAPDH was taken as an internal control. Data represent the mean of three replicate experiments.(*p < 0.05, **p < 0.01, ***p < 0.001).

Since IBRV infection increased the expression of bta-miR-146b, we hypothesized that bta-miR-146b might affect IBRV replication in host cells. To verify this hypothesis, we upregulated and knocked down bta-miR-146b expression by utilizing its mimics and inhibitor and evaluated their effects on IBRV replication. As shown in **Figure 2A**, bta-miR-146b mimics or inhibitor could effectively upregulate or knockdown endogenous bta-miR-146b expression. Then we determined the IBRV growth in MDBK cells transfected with bta-miR-146b mimic or inhibitor at different time points (12, 24, 48 h) after IBRV infection using the TCID_50_ method. The results showed that overexpression of bta-miR-146b in MDBK cells significantly enhanced IBRV replication in the cells compared to controls (**Figure 2B**), and conversely knockdown of endogenous bta-miR-146b in MDBK cells significantly inhibited IBRV replication (**Figure 2C**). In addition, we found that the up-regulation of IBRV gD expression upon overexpression of bta-miR-146b and the suppression of IBRV gD expression upon knockdown of bta-miR-146b were also confirmed by qRT-PCR (**Figure 2D**) and western blot (**Figure 2E**). These data clearly indicate that bta-miR-146b promotes IBRV replication in cells and is an important regulator of IBRV infection.

### 3.3 bta-miR-146b suppresses type I interferon expression in MDBK cells

**Figure 3:**
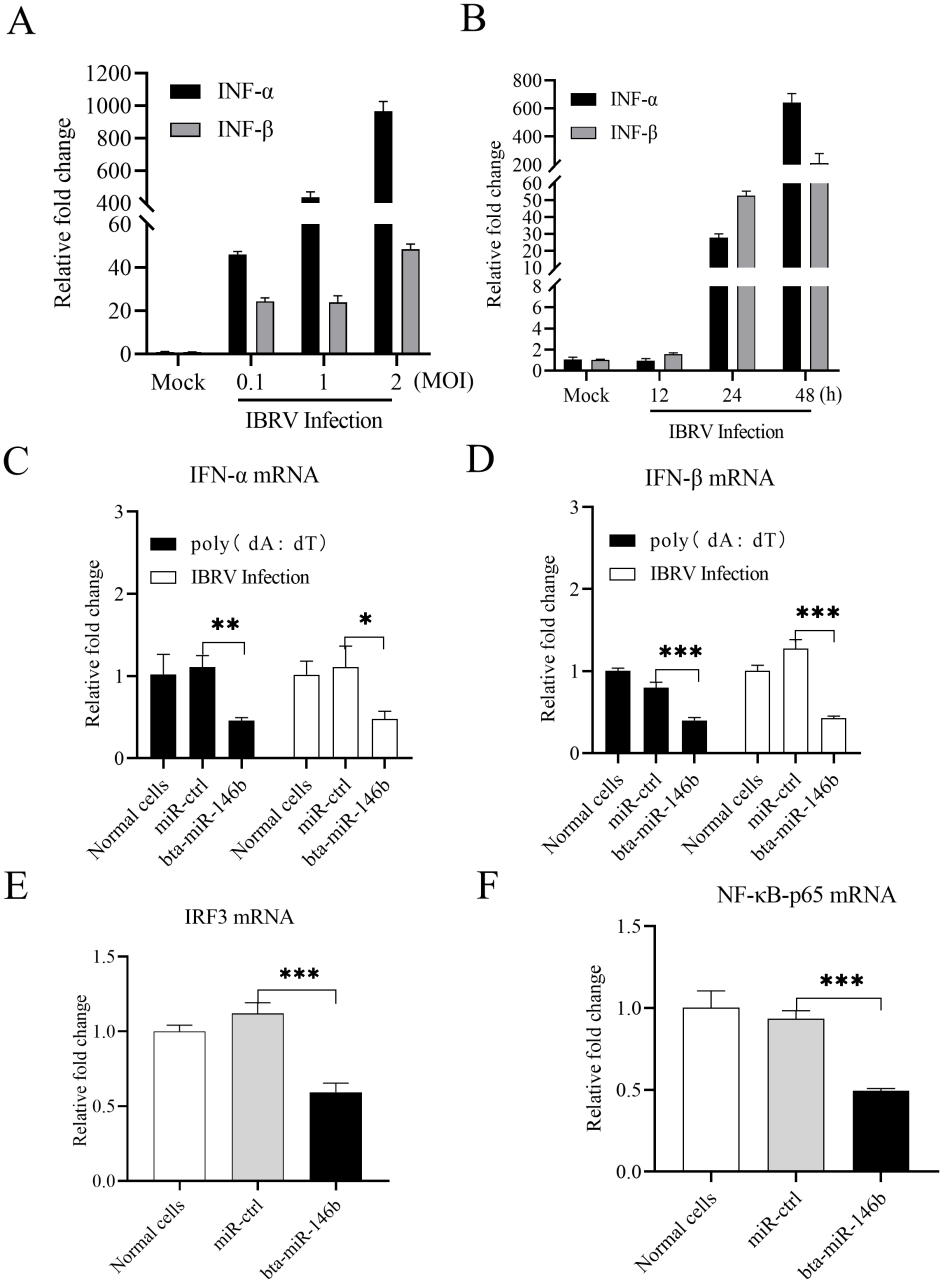
bta-miR-146b suppresses IBRV-induced type I interferon expression in MDBK cells. **(A)** Measurement of type I interferon expression in MDBK cells after infection with different doses of IBRV. MDBK cells were infected with IBRV at MOIs of 0.1, 1 and 2, respectively. After 24 h of infection, total cellular RNA was extracted and qRT-PCR was performed to analyze the expression of IFN-α and IFN-β. GAPDH was used as an internal control. **(B)** Examination of type I interferon expression at different time points after IBRV infection in MDBK cells. MDBK cells were infected with IBRV with an MOI of 1. At different time points of IBRV infection (12, 24, 48 h), expression of type I interferon was analyzed by qRT-PCR as above. GAPDH was used as an internal control. **(C, D)** MDBK cells were transfected with miRNA controls or bta-miR-146b mimics, then the cells were infected with IBRV at an MOI of 1 or treated with poly (dA:dT) at a final concentration of 2 μg/mL at 24 h post transfection. The cellular total RNA was extracted after 24 h of IBRV infection or poly (dA:dT) treatment RNA and performed to detect the expression of IFN-α and IFN-β. GAPDH was used as an internal control. **(E, F)** MDBK cells were transfected with miRNA controls or bta-miR-146b mimics, at 24 h post transfection, the cells were treated with poly(dA:dT) for 24 (h) Total cellular RNA was extracted, and qRT-PCR was performed to detect the expression of IRF3 **(E)** and p65 **(F)**. GAPDH was used as an internal control. Data represent the mean of three replicate experiments. (*p < 0.05, **p < 0.01, ***p < 0.001).

To investigate whether bta-miR-146b’s regulation of IBRV replication in MDBK cells is related to type I interferon expression, we examined type I interferon expression in IBRV-infected MDBK cells first. We found that the expression of type I interferons (IFN-α, IFN-β) was significantly induced in cells after IBRV infection in a dose-dependent (**Figure 3A**) and time-dependent (**Figure 3B**) manner. We then overexpressed and knocked down bta-miR-146b expression utilizing its mimics and inhibitor and determined their effects on cellular type I interferon expression. We transfected MDBK cells with bta-miR-146b mimics and examined the expression of type I interferon in these cells after poly(dA:dT) treatment or IBRV infection. The results showed that overexpression of bta-miR-146b significantly suppressed the expression of type I interferon in IBRV-infected or poly(dA:dT)-treated cells (**Figures 3C, D**). Further, we examined two important transcriptional regulators of type I interferon expression: IRF3 and p65, a member of the NF-κB family. The results showed that overexpression of bta-miR-146b also significantly inhibited the expression of IRF3 and p65 in poly(dA:dT)-treated cells (**Figures 3E, F**). These data suggest that bta-miR-146b inhibits type I interferon and IRF3 expressions in cell response to DNA virus infection.

### 3.4 bta-miR-146b directly targets IRAK1 in MDBK cells

**Figure 4:**
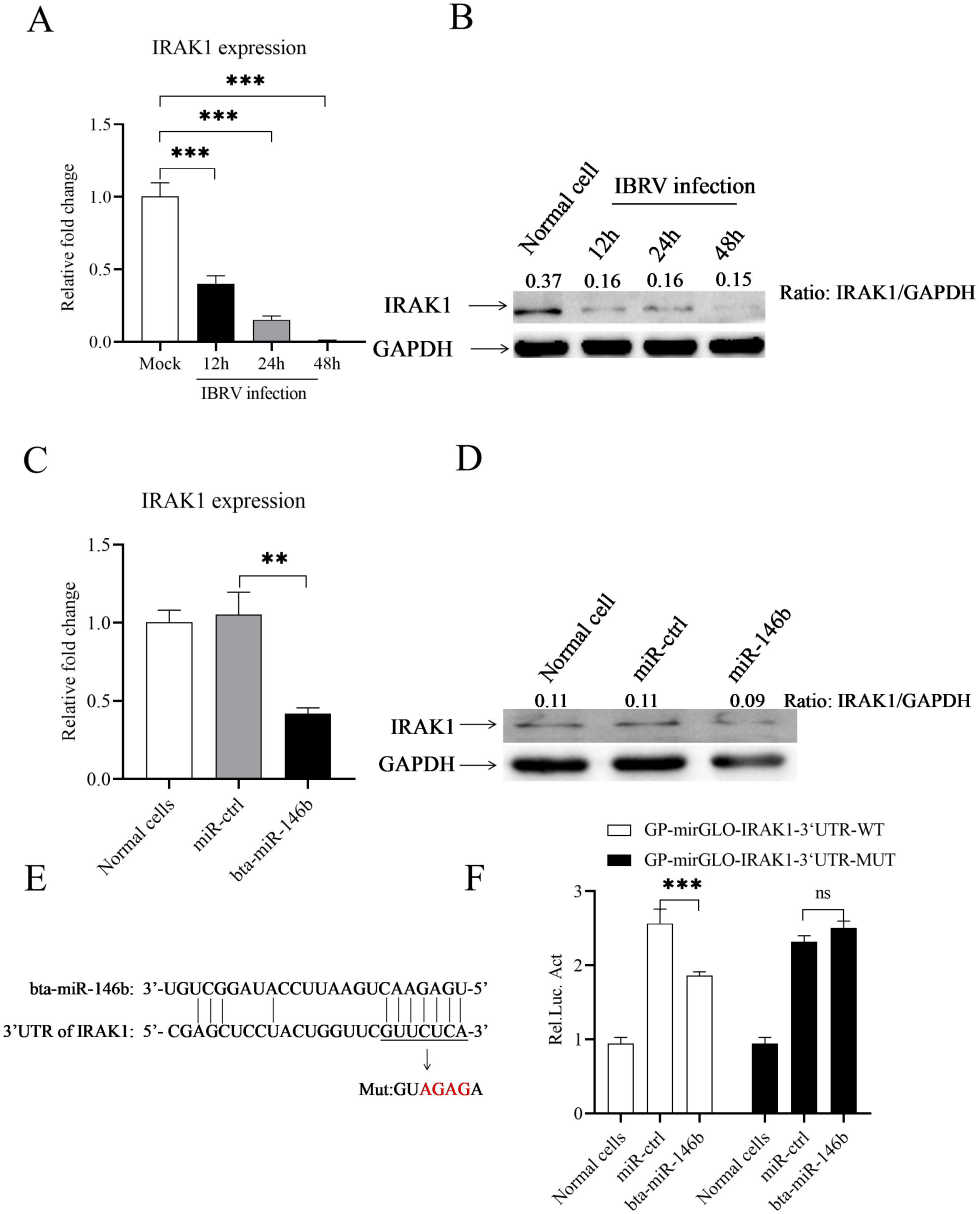
IRAK1 is a direct target gene of bta-miR-146b. **(A, B)** IBRV infection inhibited IRAK1 expression in host cells. MDBK cells were infected with IBRV at an MOI of 1. Total RNA of MDBK cells was extracted at different time points (12, 24, 48 h), and qRT-PCR was performed to detect the expression of the IRAK1 gene. GAPDH was used as an internal control **(A)**. Cell lysates were prepared at different time points of IBRV infection for western blot to detect IRAK1 protein expression, and the expression of endogenous GAPDH was taken as an internal control **(B-D)** Overexpression of bta-miR-146b suppressed IRAK1 expression in MDBK cells. MDBK cells were transfected with bta-miR-146b mimics or miRNA controls, and qRT-PCR was performed to detect IRAK1 mRNA expression at 24 h post-transfection. GAPDH was used as an internal control **(C)**. Cell lysates were prepared at 24 h post-transfection and performed for western blot to detect IRAK1 protein expression **(D, E)** Scheme of the predicted target site of bta-miR-146b in the IRAK1 gene. bta-miR-146b seed sequences are underlined and mutated as indicated by the arrows. **(F)** HEK 293T cells were cotransfected with bta-miR-146b mimics or miRNA controls together with luciferase reporter gene vectors, and after 48 h of transfection, the cells were lysed and luciferase activity was determined. The relative level of luciferase activity (Rel Luc Act) was calculated as follows: (luciferase activity of cells co-transfected with reporter gene plasmid and bta-miR-146b mimics)/(luciferase activity of cells co-transfected with reporter gene plasmid and miRNA controls). Data represent the mean of three replicate experiments. (**p < 0.01, ***p < 0.001).

Using TargetScan and miRanda prediction software, we found that IRAK1, the predicted target gene of bta-miR-146b, may regulate type I interferon signaling in MDBK cells. To verify this hypothesis, we first examined the expression of IRAK1 during IBRV infection, and the results showed (**Figures 4A, B**) that the expression of IRAK1 was significantly suppressed during IBRV infection. Furthermore, we analyzed the effect of bta-miR-146b on IRAK1 expression, and we found that overexpression of bta-miR-146b by transfection of its mimics significantly suppressed the expression of IRAK1 (**Figures 4C, D**). After that, we utilized luciferase assays to search for direct evidence of the interaction between bta-miR-146b and IRAK1. We constructed the dual-luciferase reporter gene plasmid GP-mirGLO-IRAK1-3’UTR-WT containing the 3’UTR around the predicted target site from IRAK1 and the mutant plasmid GP-mirGLO-IRAK1-3’UTR-MUT with a mutation in the seed region, respectively (**Figure 4E**). The effect of bta-miR-146b on reporter gene activity was tested using a dual luciferase reporter gene assay. The results showed (**Figure 4F**) that bta-miR-146b significantly inhibited the luciferase activity of GP-mirGLO-IRAK1-3’UTR-WT but did not affect the luciferase activity of GP-mirGLO-IRAK1-3’UTR-MUT, as compared with the control. These data strongly suggest that bta-miR-146b represses IRAK1 expression by sequence-specific binding to the 3’ UTR of the IRAK1 gene.

### 3.5 Knockdown of IRAK1 rescues bta-miR-146b inhibitor-induced inhibition of IBRV replication

**Figure 5:**
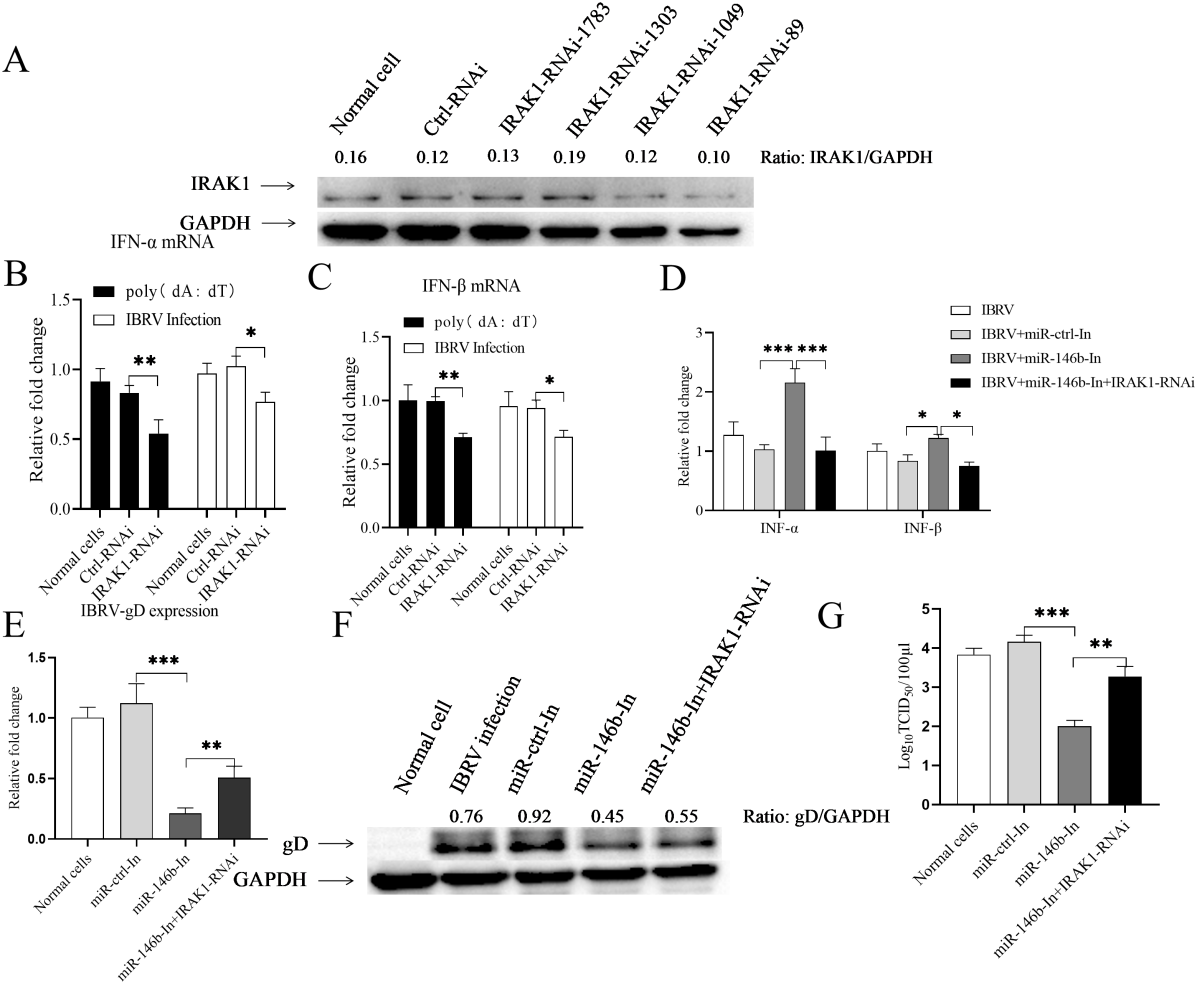
Knockdown of IRAK1 restored bta-miR-146b inhibited IBRV replication. **(A)** Effect of IRAK1 RNAi on endogenous IRAK1 expression in MDBK cells. MDBK cells were transfected with siRNA constructs (IRAK1-siRNA-1783/1303/1049/89) or controls. Twenty-four hours after transfection, cell lysates were prepared, and IRAK1 expression was assayed by western blot with endogenous GAPDH expression as an internal control. **(B, C)** Examination of the expression of type I interferon in poly (dA:dT) treated, or IBRV infected MDBK cells after IRAK1 knockdown. MDBK cells were transfected with siRNA or siRNA control. Twenty-four hours after transfection, cells were infected with IBRV at an MOI of 1 or treated with poly (dA:dT) at a final concentration of 2 μg/ml. Twenty-four hours after infection or poly (dA:dT) treatment, total RNA was extracted, and qRT-PCR was used to detect the expression of IFN-α **(B)** and IFN-β **(C)**. GAPDH was used as an internal control. **(D)** Knockdown of IRAK1 restores type I interferon expression inhibited by bta-miR-146b. MDBK cells were transfected with bta-miR-146b inhibitor alone or co-transfected with IRAK1-siRNA, and qRT - PCR was performed to detect the expression of IFN-α and IFN-β at 24 h post-transfection. GAPDH was used as an internal control. **(E, F)** Knockdown of IRAK1 restores gD gene expression suppressed by bta-miR-146b. MDBK cells were transfected with bta-miR-146b inhibitor alone or co-transfected with IRAK1-siRNA, and MDBK cells were infected with IBRV at an MOI of 1 at 24 h post-transfection, gD expression was analyzed by qRT - PCR or western blot. **(G)** Knockdown of IRAK1 restores IBRV replication inhibited by bta-miR-146b. MDBK cells were transfected with bta-miR-146b inhibitor alone or co-transfected with IRAK1-siRNA, and MDBK cells were infected with IBRV at an MOI of 1 after 24 h of transfection, the TCID_50_ assay was used to determine the viral titer in cell cultures at 48 d.p.i. Data represent the mean of three replicate experiments. (*p < 0.05, **p < 0.01, ***p < 0.001).

To examine the role of IRAK1 in the regulation of viral replication by bta-miR-146b, we designed four IRAK1 RNAi constructs, and we found that IRAK1-RNAi-89 could effectively lower the cellular level of IRAK1 without causing discernible changes in cell viability (**Figure 5A**). Then, we transfected MDBK cells with siRNA or siRNA control and detected the expression of type I interferon in cells after poly (dA:dT) treatment or IBRV infection. The results showed that IRAK1 knockdown by RNAi significantly inhibited the expression of type I interferon in MDBK cells with either IBRV infection or poly (dA:dT) treatment (**Figures 5B, C**). We co-transfected MDBK cells with the bta-miR-146b inhibitor and IRAK1 RNAi and then infected them with IBRV at 24 hours post-transfection and examined the effect of IRAK1 knockdown on the promotion of type I interferon expression by the bta-miR-146b inhibitor. The results showed that the enhanced expression of IFN-α and IFN-β induced by the bta-miR-146b inhibitor was blocked by IRAK1 silencing (**Figure 5D**), indicating that bta-miR-146b regulates type I interferon expression through IRAK1.

We then examined the IBRV growth in these cells receiving cotraninfection of siRNA and bta-miR-146b inhibitor. The data showed that the bta-miR-146b inhibitor caused inhibition of IBRV replication in MDBK cells were rescued (**Figures 5E-G**), indicating that bta-miR-146b regulates IBRV replication in MDBK cells through IRAK1.

## 4 Discussion

IBRV infection can cause severe respiratory infections in cattle, and it has the ability to disrupt the host’s immune system, increasing the chance of secondary infections (Rimayanti et al., 2024). It has been demonstrated that IBRV can target the cellular interferon pathway to regulate the immune response (Jones, 2009), however, studies on miRNAs and IBRV infection are still limited. In this study, we determined that bta-miR-146b was upregulated during IBRV infection in MDBK cells. Further, bta-miR-146b could regulate IBRV replication and host type I interferon production. Moreover, we found that the regulatory role of bta-miR-146b was achieved by targeting the IRAK1 gene. These findings suggest that bta-miR-146b plays significant roles in IBRV infection and viral immune escape.

It has been widely established that miRNAs can regulate viral replication by targeting the viral genome or modulating intrinsic or adaptive immunity (Bernier and Sagan, 2018). Certain viruses can directly encode miRNAs to exert regulatory effects (Diggins and Hancock, 2023) and on the other hand, viruses can modulate the infection process by regulating host miRNAs (Zhuo et al., 2013). In this study, we utilized a deep sequencing method to assay miRNA changes in IBRV infected MDBK cells and found that up to 181 miRNAs were altered in expression, among which miR-146b was determined to be upregulated after IBRV infection and was demonstrated to be able to promote IBRV replication as a pro-infection factor.

miR-146b has been reported to be involved in a variety of human disease processes, including cancer (Kanokudom et al., 2018) and miscarriage (He et al., 2023). The molecule has been reported to be associated with autophagy, apoptosis, proliferation and inflammatory responses in some research (Zhang et al., 2023). In recent years, miR-146b has also been found to regulate viral replication processes, for example, miR-146 has been reported to promote the replication of Tembusu virus (Chou et al., 2017) and Severe Fever With Thrombocytopenia Syndrome Virus (Huang et al., 2023), the mechanism involves direct targeting of Ribosomal protein S14 (RPS14), or regulation of differentiation of macrophages into M2 cells by targeting STAT1. Although the direct regulatory relationship between miR-146b and type I interferon has not been demonstrated by any study, its ability to modulate host immune responses has been confirmed (Zhang et al., 2021). By targeting IFI35, miR-146b suppresses lipopolysaccharide-induced inflammation and apoptosis in glomerular cells through the JAK1/STAT1 signaling pathway. Mice deficient in miR-146b exhibit a weaker neuroinflammatory response upon LPS stimulation (Chithanathan et al., 2022).Additionally, miR-146b has been demonstrated to participate in regulating the polarization of macrophages (Peng et al., 2016). In this study, we found that miR-146b regulates IBRV replication by interfering with type I interferon.

Type I interferon plays critical functions in host anti-infection immunity and serves as the host’s first line of defense against viral infections. Many miRNAs are able to regulate type I interferon expression by targeting key molecules of the type I interferon signaling pathway (Zhang et al., 2019). miR-155 has been reported to enhance type I interferon expression and suppressed IBDV replication via targeting SOCS1 and TANK (Forster et al., 2015), and miR-677 regulates CPIV3 replication by targeting the MAVS mitochondrial antiviral signaling protein (MAVS) (Wang et al., 2018). In this study, we found that the enhanced type I interferon expression caused by knockdown of bta-mir-146b could be rescued by silencing interleukin-1 receptor-associated kinase (IRAK1) expression, suggesting that the regulatory effect of mir-146b on type I interferon is mediated by IRAK1.

IRAK is a serine-threonine kinase present in host cells. This protein is a key node in the TLRS and IL-1RS signaling pathways and is involved in host innate immunity by regulating host inflammatory gene expression (Zhong et al., 2022). The protein has been reported to be extensively involved in host diseases such as inflammatory disease, autoimmune disease, cardiovascular disease, neurodegenerative diseases and even multiple types of cancers (Gottipati et al., 2008). The involvement of IRAK1 in viral infections has also been confirmed, which may be due to its ability to participate in the expression of interferon (Kim et al., 2024) and IL17 (An et al., 2008). A paper published in 2023 reported that PPRV-induced novel miR-3 contributes to inhibiting type I IFN production by targeting IRAK1 (Sun et al., 2018). In the present study, we also found that knockdown of IRAk1 expression inhibited poly(dA:dT) induced type I IFN production in MDBK cells, indicating the close relationship between IRAK1 and type I interferon signaling pathway.

As a powerful protein, IRAK1 is regulated by a variety of miRNAs and is involved in a wide range of disease processes. The relationship between IRAK1 and miR-146b has been demonstrated in many types of cancers (Chou et al., 2016; Li et al., 2021). One study found that miR-146b-5p enhances the sensitivity of NSCLC to EGFR tyrosine kinase inhibitors by regulating the IRAK1/NF-κB pathway (Abolfathi et al., 2023), and in the present study we found that IRAK1 was the direct target gene of bta-miR-146b by the luciferase assay. IRAK1 is a key regulator in the type I interferon pathway, and its downregulation directly leads to the suppression of type I interferon expression. In this study, we found that knockdown of IRAK1 via silencing blocked the upregulation of type I IFN expression induced by bta-miR-146b inhibitor. This suggests that IRAK1 plays a crucial role in the regulation of miR-146b, which in turn affects type I IFN. This finding is consistent with previous reports identifying IRAK1 as a regulator of IFN expression (Liu et al., 2020).

Of course, there are still some questions that need to be addressed in this study. For example, multiple viral proteins of IBRV are involved in the regulation of IFN production, and which one is associated with the up-regulated expression of bta-miR-146b? Does miR-146b work similarly on other viruses? In addition to miR-146b, we also discovered numerous significantly differentially expressed miRNAs. Whether these miRNAs are also associated with IBRV replication and play important roles remains to be further investigated.

In summary, we present a strategy used by IBRV to escape innate immunity by engaging miRNA, which may help us to further understand IBRV pathogenesis. We found that bta-miR-146b inhibits type I IFN signaling and promotes IBRV replication by targeting IRAK1, a critical signaling mediator of innate immunity. These findings have provided insights for further studies of the molecular mechanism underlying host response to IBRV infection.

## Data Availability

The datasets presented in this study can be found in online repositories. The data that support the findings of this study are openly available in Science Data Bank. The DOI is 10.57760/sciencedb.27253.
